# The Use of Chitosan and Starch-Based Flocculants for Filter Backwash Water Treatment

**DOI:** 10.3390/ma15031056

**Published:** 2022-01-29

**Authors:** Piotr Maćczak, Halina Kaczmarek, Marta Ziegler-Borowska, Katarzyna Węgrzynowska-Drzymalska, Aleksandra Burkowska-But

**Affiliations:** 1 Faculty of Chemistry, Nicolaus Copernicus University in Toruń, Gagarina 7, 87-100 Torun, Poland; martaz@umk.pl (M.Z.-B.); kasiawd@doktorant.umk.pl (K.W.-D.); 2 Water Supply and Sewage Enterprise LLC, Przemysłowa 4, 99-300 Kutno, Poland; 3 Faculty of Biological and Veterinary Sciences, Nicolaus Copernicus University in Toruń, Lwowska 1, 87-100 Torun, Poland; wodkow@umk.pl

**Keywords:** chitosan, starch, dialdehyde biopolymers, flocculants, water treatment, biodegradation

## Abstract

Inorganic aluminum or iron salts supported with synthetic polymers are commonly used to eradicate colloidal particles from water in coagulation and flocculation processes. Nevertheless, these agents have several disadvantages, such as large volumes of sludge produced or environmental toxicity. Recently biodegradable polymers have been suggested as eco-friendly flocculants for water treatment. This study aimed to investigate the possibilities of using starch and chitosan and their oxidized derivatives as flocculants for filter backwash water treatment. Dialdehyde starch (DST) and dialdehyde chitosan (DCT) were synthesized by periodate oxidization of natural starch from corn and low molecular weight chitosan. The obtained materials have been characterized with scanning electron microscopy (SEM), ATR-FTIR spectroscopy, and thermogravimetric analysis (TGA). Furthermore, we studied the flocculation properties of polysaccharide flocculants in a series of jar tests. The effectiveness of chitosan and starched-based flocculants was compared to synthetic polymers commonly used to treat iron ions-rich filter backwash water. The environmental aspects of these chemicals, particularly the biodegradability of post-flocculation residues, were also addressed. It was found that oxidized starch and chitosan derivatives can be used as ecological flocculating materials to treat potable water or sludge.

## 1. Introduction

Nowadays, water purification in safe drinking water production is one of the most critical problems undertaken by scientists [1,2,3,4]. The most frequently used process in classic water treatment systems is filtration [5]. Unfortunately, this creates a lot of so-called filter backwash water (FBW), containing colloidal contaminants washed out from the filter bed. FBW is treated in a technological process using an aggregation mechanism to minimize wastewater production. In practice, coagulation and flocculation are now commonly used processes in water treatment [6]. These techniques facilitate removing fine particles suspended in the liquid by forming well-defined aggregates that can quickly settle out from the water.

Coagulation is a phenomenon of deposition of colloid particles induced by the addition of electrolytes, which most often is aluminum or iron compound, generally referred to as a coagulant [7,8]. These chemical agents help remove the naturally occurring pollutants by their hydrolysis and oxidation to compounds sparingly soluble in water, leading to the formation of agglomerates. The next step is flocculation, the process of creating larger agglomerates (i.e., flocs) [7,9,10]. The choice of an effective coagulant is determined primarily by its ability to bind colloidal impurities and create durable, insoluble systems that facilitate their mechanical removal from water. However, the electrolyte-induced sedimentation rate is relatively low.

**Figure 1:**
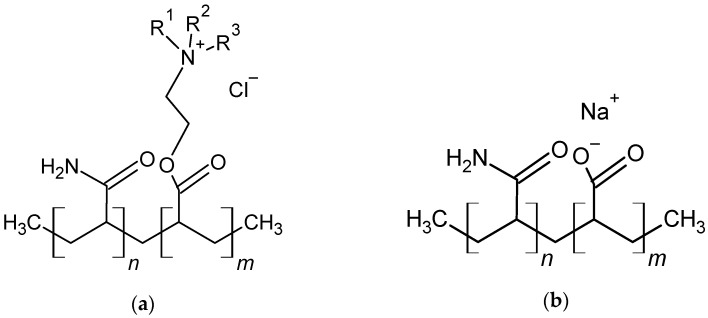
Chemical structure of cationic polyacrylamide (**a**) and anionic polyacrylamide (**b**) commonly used in water purification.

Therefore, a small amount of polymer is added to increase settlement speed in the colloidal aggregation [8]. Such compounds, called flocculants or floc agents, are most often anionic or cationic polyacrylamides (Figure 1) [11,12]. Synthetic polymers are characterized by a high ability to bind impurities and form dense aggregates.

Nonetheless, the main disadvantage of these agents is environmental pollution. Carcinogenic and neurotoxic monomer residue in polyacrylamides pollute water and pose a potential risk to human health [13]. Therefore, an equally important aspect, as the ability to aggregate contaminants, is to minimize the negative impact of the used coagulants and polyelectrolytes on the treated water parameters. Accordingly, it is vital to determine the flocculants’ effectiveness and their effect on water quality. Recently, some scientific works have focused on using polysaccharides as eco-friendly materials to improve the coagulation/flocculation processes [14,15,16,17,18,19,20,21]. An excellent example may be chitosan and starch, renewable polymers of proven biocompatibility and non-toxicity [22].

The application of these biopolymers in various industrial branches increases due to their effective chemical modification leading to the desired changes in utility properties. The most common reactions used for this purpose include etherification, esterification, cross-linking, grafting, complexation and oxidation. The latter modification type, carried out under appropriate conditions, enriches the macromolecules with aldehyde groups, contributing to improving water solubility and increasing adhesion to other compounds. The use of periodate as an oxidizing agent leads to selective oxidation and opening the glycoside ring. Moreover, oxidation changes molecular organization—it usually reduces the ordering. Possibilities of various applications of dialdehyde polysaccharides as functional materials have recently been reviewed by Ding and Wu [23].

**Figure 2:**
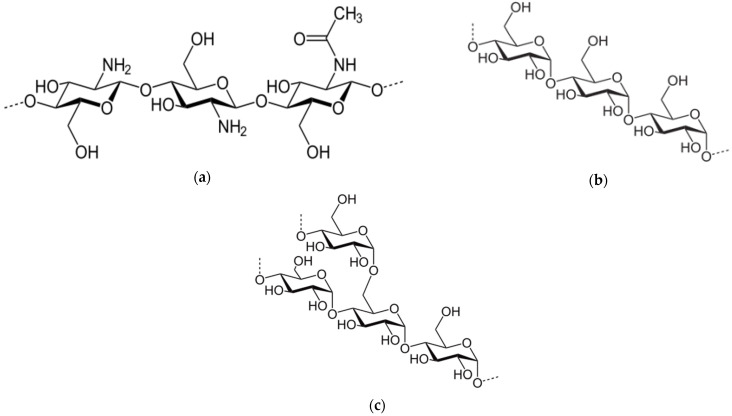
Chemical structure of chitosan copolymer (**a**), and components of starch: amylose (**b**) and amylopectin (**c**).

Chitosan (CT) is a partially deacetylated derivative of chitin—a fundamental constituent of crustaceans shells [24]. It is a linear copolymer of D-glucosamine and N-acetyl-d-glucosamine (Figure 2a) [7]. Grafted chitosan copolymers are recommended as an eco-friendly flocculant for water and wastewater treatment with acrylamide [17,25,26,27]. Moreover, chitosan and its derivatives are known for their biocidal properties, valuable in biomedical applications.

Another example of a promising bio-based flocculant is starch. It is one of the most abundant biopolymers in nature, extracted mainly from potatoes, corn, or rice. The starch macromolecule consists of linear amylose (Figure 2b) and branched amylopectin (Figure 2c) [28]. Similar to chitosan, starch is used as a flocculant in the form of copolymers, mostly with polyacrylamide [29,30]. 

Charge density, expressed as a percentage of ions fractions containment, plays a considerable role in the flocculation process. Polyacrylamides commonly used in water treatment contain carboxyl groups (-COOH), dissociating at a high pH value, giving the polymer a negative charge. Moreover, amides (-CONH_2_) groups can also be hydrolyzed into carboxyl groups so that both active (carboxyl and amide) sites increase electrostatic attraction to the oppositely charged colloidal particles and enhance aggregation. Furthermore, those groups can create hydrogen bonds with mineral-organic complexes present in the water [31]. Similarly, the presence of reactive hydroxyl (-OH) functional groups in the structure of starch, as well as amino groups (-NH_2_) in the chitosan backbone, enhances their adsorption properties creating hydrogen bonds [2]. Moreover, chitosan can chelate metal ions due to active sites from two adjacent macromolecules [32].

Therefore, polysaccharides may bring measurable benefits by improving the efficiency of the coagulation/flocculation process.These non-toxic and biodegradable biopolymers reduce the negative impact on the environment compared to synthetic polyelectrolytes and significantly increase the health safety of the drinking water.

The purpose of this work was to synthesize polysaccharide-based flocculants: dialdehyde starch (DST) and dialdehyde chitosan (DCT) and further evaluate their properties in the flocculation process. The chemical structure, morphology, thermal, and biodegradation properties of oxidized biopolymers were characterized. The leading goal was to test the effectiveness of obtained biopolymers as filter backwash water (FBW) treatment flocculants and compare them with the performance of unmodified polysaccharides and the selected commercial materials used in practice.

## 2. Materials and Methods

### 2.1. Materials

Chitosan (low molecular weight) 75–85% deacetylated (CAS 9012-76-4) and starch from corn containing approximately 73% amylopectin and 27% amylose (CAS 9005-25-8) were purchased from Sigma–Aldrich (Munich, Germany). Acetic acid and acetone of reagent grade were obtained from Avantor Performance Materials Poland S.A. (Gliwice, Poland). Sodium periodate was purchased from Sigma–Aldrich (Munich, Germany). Synthesis and characteristics of bio-based flocculants were performed at the Faculty of Chemistry and Faculty of Biological and Veterinary Sciences, Nicolaus Copernicus University (NCU) in Toruń. The flocculation jar tests were carried out at the Water Treatment Plant (WTP) in Kutno, Water Supply and Sewage Enterprise LLC.

### 2.2. Water Samples

Water samples were collected at the Water Treatment Plant (WTP) in Kutno, Poland. Samples were typical filter backwash water (FBW) contaminated mainly with iron compounds that affect their high turbidity. Total iron ions concentration and turbidity value are parameters that considerably impact the quality of water. The reduction of these parameters has been recognized as a determinant of flocculation efficacy.

### 2.3. Coagulant

The effectiveness of native and oxidized polysaccharides as flocculants in optimal dosage was evaluated with the assistance of hydrolyzed polyaluminum chloride PAX XL10 in the amount of 1 mg Al^3+^/L as a primary coagulant. The dose was selected based on operating experience treating FBW at WTP in Kutno, Poland.

### 2.4. Polymer Flocculants

Eight currently-used polyacrylamide flocculants with different structures (cationic, anionic, and non-ionic) were chosen for comparative studies. Synthetic polymers (commercially available as Superfloc or Optifloc—see Table 1 below) were products of Kemira Company (Helsinki, Finland). Samples were provided courtesy of Best-Chem (Sochaczew, Poland).

Five hundred mg of selected polyacrylamides were placed in a 500-mL volumetric flask and diluted with tap water resulting in a 1 mg/L stoke solution.500 mg of dry corn starch and polysaccharides oxidized derivatives were also dispersed in 500 mL of tap water by rapidly mixing for 1 h to prepare stock solutions. The exact amount of chitosan was dissolved in 500 mL of 0.1 M HCl, resulting in 1 mg of polymer per mL solution.

### 2.5. Synthesis of DST and DCT

The dialdehyde derivatives of starch (DST) and chitosan (DCT) were obtained by oxidation with sodium periodate (NaIO_4_). Three g of corn starch were dissolved in 60 mL distilled water, mixed with the appropriate quantity of sodium periodate (0.7 M, 20 mL), and blended using a magnetic stirrer at 40 °C for three h. When the reaction solution had cooled, products were precipitated by adding acetone and isolated by filtration. Finally, the sediments were washed three times with a water-acetone (40/60 *v*/*v*) mixture and dried at room temperature for 24 h. For DCT synthesis, three grams of chitosan were dissolved in a 300 mL 1% acetic acid solution by mechanical stirring for 12 h. Oxidation was carried out according to the procedure mentioned above for DST.

### 2.6. Jar Tests and Flocculation Properties Evaluation

Jar tests were conducted using a digital jar test apparatus—flocculator JLT6 VELP Scientifica for each polymer flocculant. The experiment simulated practical procedures used as a standard at WTP in Kutno.

The addition of the polymers was followed by rapid mixing (250 rpm) for 1 min and slow mixing (25 rpm) for 15 min. After another 30 min of settling, the samples were taken from each jar for further analysis. For the iron determination, the colorimetric method with 1,10-phenanthroline was carried out on a DR1900 Portable Spectrophotometer, according to the Hach Method 8008 [33] approved by the United States Environmental Protection Agency (USEPA). The turbidity was measured using the nephelometric method on 2100 Q Portable Turbidimeter coherent with USEPA Method 180.1 [34].

### 2.7. Attenuated Total Reflectance Fourier Transform Infrared (ATR-FTIR) Spectroscopy

The Fourier transform infrared (FTIR) spectroscopy was used to identify specific chemical groups in the materials. FTIR spectra of chitosan, starch, and dialdehyde derivatives have been obtained on a Perkin Elmer Spectrum Two^TM^ FT-IR Spectrometer (Perkin Elmer, Waltham, MA, USA) with an ATR tool equipped with a diamond crystal. The scanning range was 400–4000 cm^−1^ and the resolution was 4 cm^−1^. In total, 32 scans were collected each time. Recorded spectra were subjected to ATR correction and normalization.

### 2.8. Scanning Electron Microscopy

The morphology of the polysaccharides flocculants was studied with the help of a scanning electron microscope (LEO Electron Microscopy Ltd. Cambridge, UK, model 1430 VP) without sputtering, using adjustable vacuum mode.

### 2.9. Thermal Analysis

The thermal properties of chitosan, starch, DST, and DCT were investigated by thermogravimetric analysis (TGA) using TA Instruments Discovery SDT 650 thermoanalyzer. The samples were heated at a constant rate of 2 °C/min up to 600 °C under a nitrogen atmosphere.

### 2.10. Biodegradation Properties

The level of metabolic activity of microorganisms participating in the biodegradation of organic compounds is an excellent indicator to determine the intensity of transformation of these compounds in the aquatic environment. The respirometric method for determining metabolic activity measures microorganisms’ biochemical oxygen demand (BOD) under strictly defined conditions. The tests were carried out using the OxiTop OC 110 measuring system, which allows for recording the results of oxygen uptake at long intervals, which is essential when testing hardly degradable compounds. The test was conducted following the manufacturer’s instructions [35].

To test oxygen consumption during the biodegradation, 250 mL of untreated water and 0.25 g of the tested flocculant were placed in the OxiTop measuring system bottles. Five drops of the nitrification inhibitor NTH 600 were added to each bottle as recommended by the manufacturer. Evolved carbon dioxide (CO_2_) was removed by natrium hydroxide from the BOD closed system. The BOD test was carried out for 14 days at 20 °C with the control sample of raw water without adding a flocculant. The results were read as oxygen consumption expressed as mg O_2_/L. The control was the water without the addition of a flocculant. The percentage degradation was calculated by comparing the BOD for a given flocculant with the theoretical oxygen demand (ThOD). The theoretical O_2_ consumption volume was calculated based on polymers’ carbon content and assuming that degraded products are entirely mineralized for CO_2_ [36].

Exactly 43.5 mL of the tested post-flocculation sludge was placed in the OxiTop measuring system bottles to test the respiratory activity of microorganisms during the biodegradation of post-flocculation sludge. Five hundred mg of mature compost (the number of bacteria in the compost was 2.3 × 10^6^ CFU/g) or 0.5 mL of activated sludge from the sewage treatment plant (30.7 × 10^6^ CFU/mL) was added to the post-flocculation sludge. One drop of the nitrification inhibitor NTH 600 was added to each bottle as recommended by the manufacturer. The BOD test was carried out for 14 days at 20 °C. The results were read as oxygen consumption expressed in mg O_2_/L and converted into oxygen consumption per g of the dry weight of post-flocculation sludge. The dry weight was determined by evaporating 25 mL of the tested post-flocculation sludge to a constant weight.

## 3. Results

### 3.1. Synthesis of Dialdehyde Derivatives

**Figure 3:**
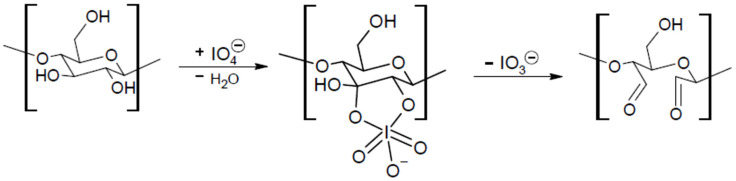
The schematic presentation of selective cleavage of the C2-C3 bond in the anhydroglucose unit (based on [37,38] and references cited therein).

The starch and chitosan derivatives were obtained by employing periodate as an oxidizing agent. During the periodate oxidation, selective cleavage of the C2–C3 bond in the anhydroglucose unit occurs, with an intermediate—a cyclic iodate diester where two aldehyde groups are formed (Figure 3) [37,38].

An equal molar ratio of polysaccharides to sodium periodate was used in both syntheses. According to our previous research, this unimolar ratio of reactants results in a high aldehyde content—33% and 58% in DST and DCT, respectively [38,39].

The obtained derivatives have been characterized by FTIR, SEM, TGA, and biodegradation tests. 

### 3.2. Characterization of Dialdehyde Chitosan (DCT) and Dialdehyde Starch (DST)

#### 3.2.1. FTIR

**Figure 4:**
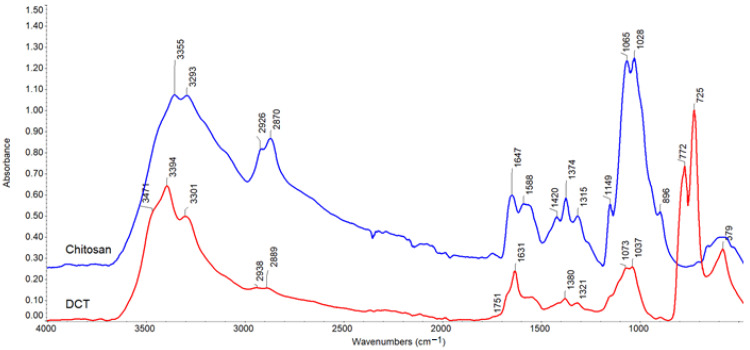
ATR-FTIR spectra for chitosan (blue) and DCT (red).

**Figure 5:**
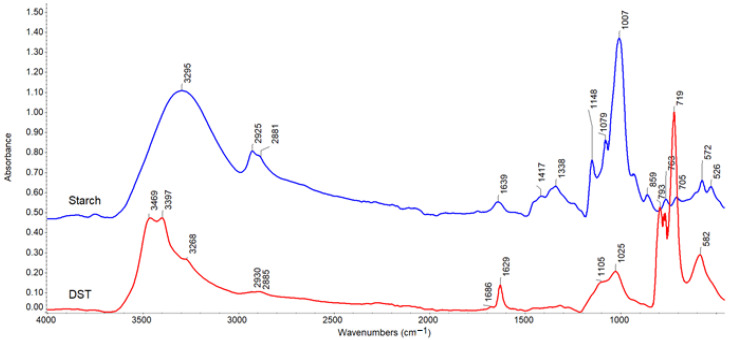
ATR-FTIR spectra for corn starch (blue) and DST (red).

The structural changes caused by the oxidation of chitosan and corn starch were confirmed by ATR-FTIR spectroscopy. The obtained spectra are compared in Figure 4 and Figure 5 below.

The characteristic absorption bands corresponding to the vibrations of the OH stretching (a broad band at 3000–3500 cm^−1^ range), CH stretching (2800–3000 cm^−1^), C-O-C (main maximum at 900–1100 cm^−1^) groups, present in the polysaccharide structure [38], were observed at the spectra of the examined compounds. Very broad hydroxyl bands indicated hydrogen-bonded OH groups. In the chitosan spectrum, bands due to N-H vibrations overlapped the OH range. In addition, amide groups absorption (amide I and II bands at 1647 and 1588 cm^−1^, respectively) is visible in the spectrum of unmodified chitosan.

**Figure 6:**
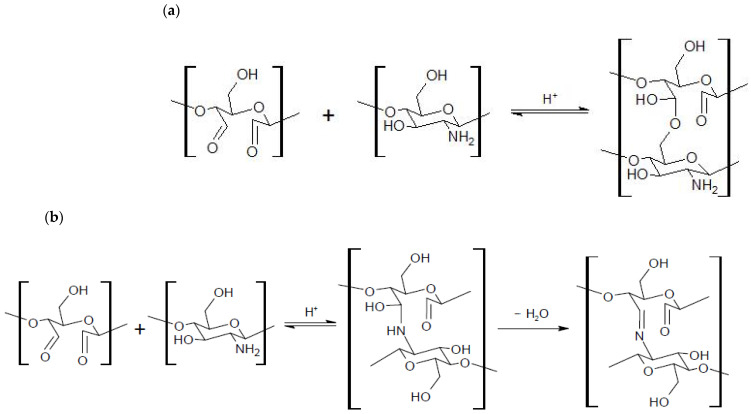
The involvement of aldehyde groups in the formation of hemiacetal (**a**) and imine (**b**) structures.

The appearance of a sharp band at 1631 cm^−1^ and 1629 cm^−1^ in DCT and DST spectrum confirmed the oxidation of polysaccharides. This band was similar to that previously described [38,39]. However, carbonyl bands at 1751 cm^−1^ (in DCT) and 1686 cm^−1^ (in DST) are very weak, which may be associated with the formation of hemiacetals [40] (Figure 6a) and, also in the case of chitosan - imine groups, i.e., Schiff’s base (Figure 6b) created in reactions with other glycoside units due to not fully oxidized polysaccharide structure.

Considerable change of shape and shift of hydroxyl bands to higher wavenumbers (the maximum at 3394 cm^−1^ in DCT and 3469, 3397 cm^−1^ in DST) proves the opening of glucoside rings.

The most significant differences have been observed in the fingerprint region attributed to C-O-C stretching overlapping with CH deformation vibrations. The strong bands with a double maximum at 1065–1028 cm^−1^ in chitosan and 1007 cm^−1^ in starch disappear. On the other hand, a new intense band arises at smaller wavenumbers (772–725 cm^−1^ in DCT and 719 cm^−1^ in DST spectrum, respectively), confirming cyclic structure opening.

The obtained results are consistent with the data in previous articles, where the IR spectra of studied polysaccharides [41,42] and their oxidized derivatives are described in detail [43,44].

#### 3.2.2. SEM Images

**Figure 7:**
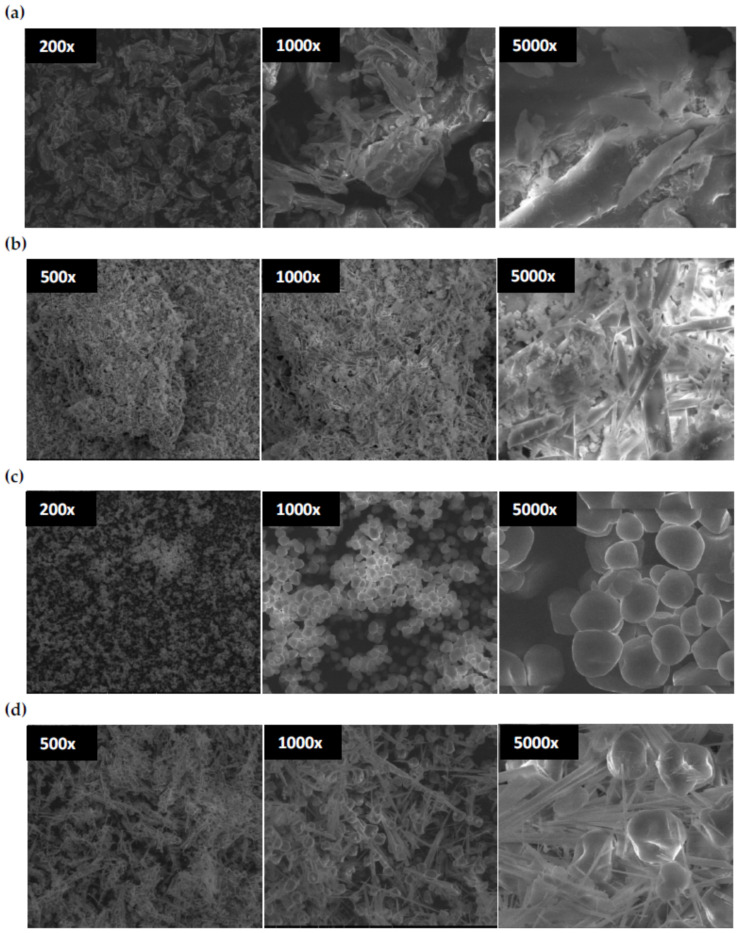
SEM images of chitosan (**a**), dialdehyde chitosan (**b**), corn starch (**c**), dialdehyde starch, (**d**) at different magnification.

Figure 7 shows the SEM images recorded for selected magnifications (from 200× to 5000×), which present the morphology typical for the tested biopolymer materials.

The native chitosan image shows irregularly shaped aggregates with sizes from 20 to 200 µm, some in the form of thick, elongated sticks (Figure 7a). The particle size distribution of this native polysaccharide is large.

Chitosan oxidation caused significant changes in morphology—numerous objects of much smaller size appear in DCT image (Figure 7b). In addition to fragmentation, a change in the shape of the particles is also observed. Mainly thin rod-shaped, spindle, and needle-like structures are formed here. Due to the fragmentation of macromolecules in DCT, the surface of the particles increases; at the same time, free spaces remain between them, which may favor the adsorption of chemical compounds from solutions.

The morphology of native corn starch is characterized by round grains with fairly regular diameters of about 10 µm (Figure 7c). As can be seen, the size spread of these spherical particles is relatively small.

In the oxidized starch (DST), a partial disappearance of the spherical structures and numerous needle forms with lengths exceeding 100 nm were observed (Figure 7d).

In both oxidized polysaccharides, somewhat similar topography was found. The elongated rod-shaped structures were formed, which may have resulted from the interaction of aldehyde groups with other functional groups in macromolecules. Moreover, the surface of spherical forms in DST became irregular due to the oxidized decomposition (C2–C3 bond break in the anhydroglucose unit) of amorphous areas in the CS structure. According to the literature [45] amorphous regions are mainly located inside starch granules. 

Degradation of corn starch due to periodate oxidation occurring in an amorphous fraction leads to deformation and, finally, to the damage of starch granules, as can be seen in Figure 7d (at the image with the 5000× magnitude). The same degradation process induced by ring-opening reaction and aldehyde groups formation results in chitosan structure fragmentation (Figure 7b).

Partially degraded macromolecules adopt different conformations, leading to elongated structures in place of the aggregates or grains dominant in the native polysaccharides. Moreover, the destruction of the crystal regions during oxidation also contributes to transforming the natural biopolymer structure.

The morphology of the tested samples may influence the flocculation properties, firstly, due to the presence of reactive groups and, secondly, due to the developed surface containing numerous active adsorption points. However, the results of flocculation studies indicate that functional groups play a significant role here.

#### 3.2.3. Thermal Analysis

**Figure 8:**
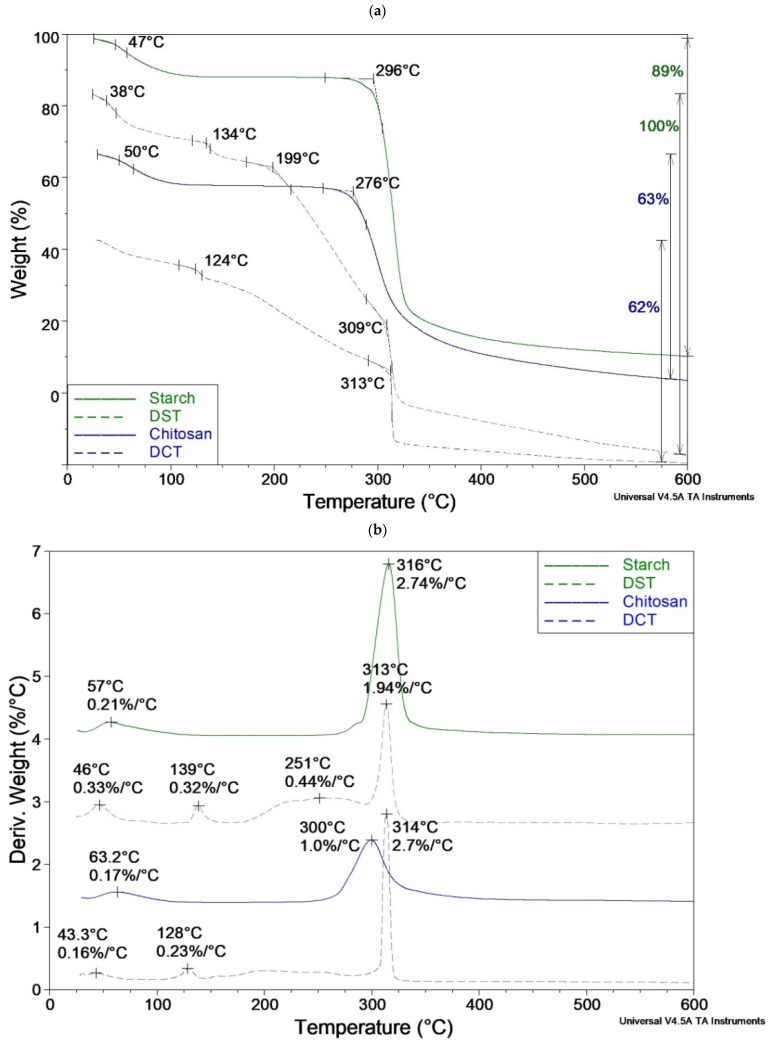

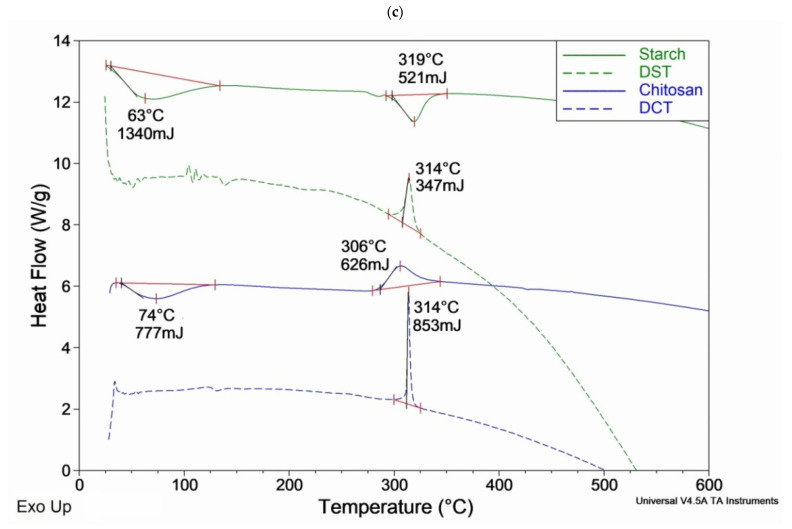
Comparison of thermogravimetric (TG, (**a**)), differential thermogravimetric (DTG, (**b**)), and heat flow (DSC, (**c**)) curves of starch, chitosan, and their oxidized derivatives (DST, DCT). The curves in figures a-c have been shifted relative to each other for clarity; the values on the scale refer to the first curve from the top (the unit is kept for all curves).

Thermogravimetric curves for native corn starch, chitosan, and their dialdehyde derivatives are shown in Figure 8. Although previous works showed changes in the thermal stability of oxidized starch and chitosan [39,40,46,47,48], specific thermal parameters have not been determined, and there is no detailed discussion and comparison with native biopolymers. TGA also provides information on the structure of the tested materials and may confirm the effectiveness of their oxidation process.

As is known, the initial weight loss in TG is related to the evaporation of adsorbed water; in our samples, it did not exceed 12% at 100 °C. The primary decomposition stage began below 300 °C in starch (T_o_ = 296 °C) and chitosan (T_o_ = 276 °C); it increased by 13–37 degrees in DST and DCT (Figure 8a, Table 2). Additional steps occurred between the first (i.e., water release) and main decomposition stages in oxidized biopolymers. The peak, with the maximum at 134 °C and 124 °C in DST and DCT, respectively (Figure 8b), could be attributed to a similar process, probably the release of strongly bound water or detachment of the weakest connected groups in structural defects. Moreover, in DST, the broad peak appears at 200–300 °C, accompanied by a 45% weight loss. In the DCT sample, a similar transformation occurred in this temperature range but was less pronounced and had a minor weight loss (about 25%). This step did not appear in native biopolymers; thus, it could be assigned to the gradual decomposition of oxidized macromolecules. Accordingly, ring-opening (selective cleavage of the C2-C3 bond) can cause thermal lability of oxidized polysaccharides, appearing as an additional step in a lower temperature range (about 200 to 300 °C). At the same time, aldehyde groups disturbed the order of macromolecules, which also contributed to their reduced thermal resistance.

The major step in thermal degradation occurred most rapidly in native starch (rate 2.74%/°C, but T_max_ is the highest 316 °C) and was slowest in chitosan (1.00%/°C, T_max_ is the lowest 300 °C).

Simultaneously measured DSC curves (heat flow) indicate an exothermic effect connected with the main decomposition step of chitosan, DCT, and DST, which could be attributed to accompanying thermal crosslinking (Figure 8c) [49]. Such crosslinked structures decomposed at higher temperatures while the experiment continued. The released heat was greatest in the DCT sample (853 mJ), at 227 mJ higher than in the original chitosan. This indicated the high cross-linking capacity of DCT (greater than in the case of DST). Only native starch exhibits an endothermic effect during thermal decomposition, which means that thermal crosslinking did not occur in this case.

Moreover, DSC curves did not show the other peaks of physical phase transitions characteristic for semicrystalline polymers (crystallization, melting), which proves the amorphous nature of the tested samples.

Total weight loss at 600 °C was lower in chitosan and DCT (63% and 62%, respectively), which indicates the higher thermal stability of crosslinked structures in these samples, contrary to starch and DST (only DST decomposes completely 100%). The main, intensive stage of decomposition of all specimens ended at approx. 350 °C; then a systematic slow weight loss was observed without visible peaks and thermal effects.

The main reactions in thermal degradation of studied polymers were side groups abstraction, ring-opening, chain scission and crosslinking. TGA results suggested that fragmentation, accompanied by the release of low-molecular-weight products, was likely to be more efficient in DCT and DST than in unoxidized CT and ST, especially in the early stages. As thermal crosslinking often led to incomplete decomposition, this process was ineffective in DST, where 100% weight loss had been observed.

### 3.3. Flocculation Study

Two series of Jar tests were carried out to evaluate the flocculation efficiency of polysaccharide-based flocculants. The first Jar tests estimated the optimal dosage of commonly used synthetic flocculants. Among the commercially available polymers, the selection was made among polyacrylamide flocculants that constituted a reference material for newly obtained flocculants. The second round assessed the flocculation efficiency of polysaccharide based materials, i.e., total iron concentration and turbidity of flocculated water. The polymers’ dosages were selected on former experience treating filter backwash water at WTP in Kutno, Poland.

**Figure 9:**
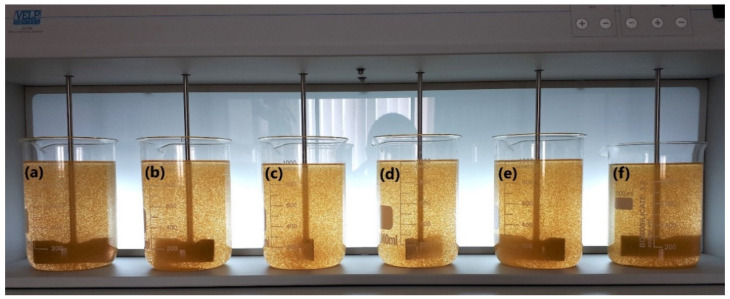
Jar test with chitozan-based flocculant in polymer dose (**a**) 0.1, (**b**) 0.2, (**c**) 0.3, (**d**) 0.5, (**e**) 1.0, (**f**) 0.2 mg/L + 1 mg Al^3+^/L of PAX XL10 coagulant, respectively.

The research compared the flocculation capability of the selected polymers and, after estimating optimal dosage, with primary coagulant PAX XL10 at the dose of 1 mg Al^3+^/L. The process was carried out on 1 L filter backwash water samples (Figure 9). After introducing the appropriate quantities of flocculants using mechanical stirrers, rapid mixing was carried out for 1 min (250 rpm), then slow mixing for 15 min (25 rpm). This was performed to simulate the conditions in the flocculation tank at the WTP. After this time, the samples were subjected to 0.5 h sedimentation. Aliquots were taken from each beaker for analysis, in which the concentration of iron ions and turbidity were determined.

The raw filter backwash water parameters changed during the research depending on the current filtration velocity. The iron ions concentration ranged from 27.55 mg/L to 33.34 mg/L, and the turbidity from 174 to 208 NTU. Table 3 summarizes the mean values of the determined parameters of the raw FBW, which were a reference point for the reduction degree calculation of the polymers’ flocculation efficiency determinants.

Results of turbidity and iron removal efficiency according to the dose of synthetic flocculant are summarized in Table 4. Almost all polymers examined in the first jar test series reached over 90% iron concentration and turbidity reduction. The most effective flocculation-promoting agent was Superfloc A150HMW—anionic polyacrylamide. In an optimal amount of 1.0 mg/L, it decreased the values of the tested water parameters by over 95%. Therefore, it has been selected to compare the flocculation efficiency of polysaccharide-based materials.

**Figure 10:**
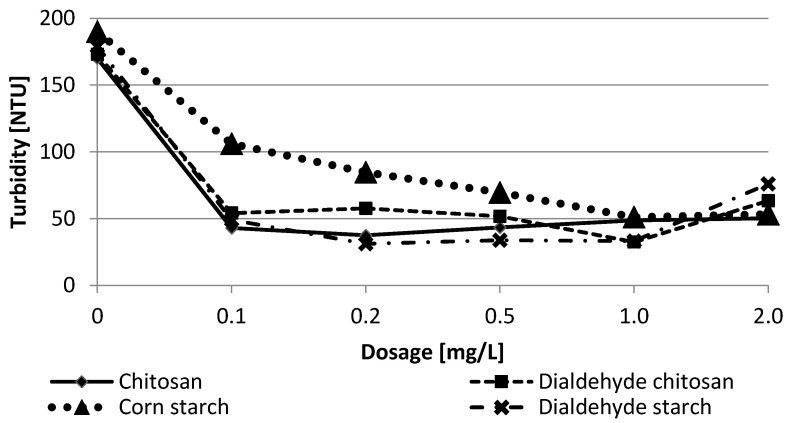
Turbidity removal according to polysaccharides dosage for filter backwash water.

**Figure 11:**
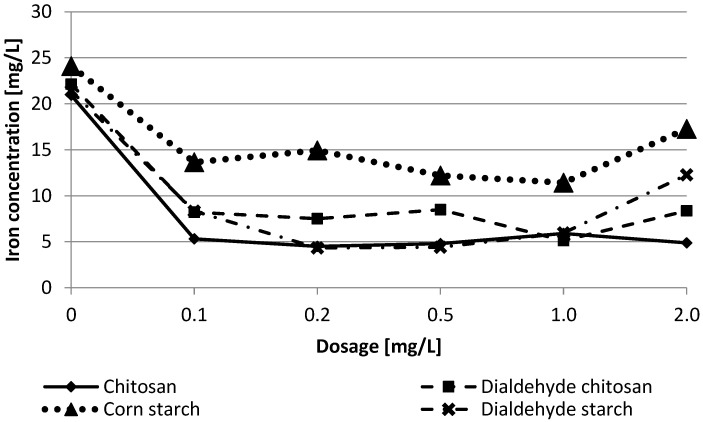
Total iron ions concentration according to polysaccharides dosage for filter backwash water.

In the next stage of trials, the flocculation capability of bio-based materials was specified. First, polysaccharides were examined in a jar test experiment to estimate the optimal dose. The test results are summarized in Figure 10 and Figure 11. As you can see, even a small dose of 0.1 mg/L resulted in more than a double reduction in the examined parameters.

In their optimal dose, tested bio-flocculants showed over 90% reduction of total iron concentration and turbidity, comparable to the polyacrylamides. The obtained results indicate that the highest water treatment efficiency was achieved for chitosan and dialdehyde starch in 0.2 mg/L. Such a small dose of the biopolymers allowed flocculation efficiency comparable to commercial flocculants. As shown in Table 5, chitosan achieved the best results in 0.2 mg/L while its dialdehyde derivative was 1.0 mg/L. Such observations indicate a high affinity of chitosan to metal ions due to its reactive amino groups.

**Figure 12:**
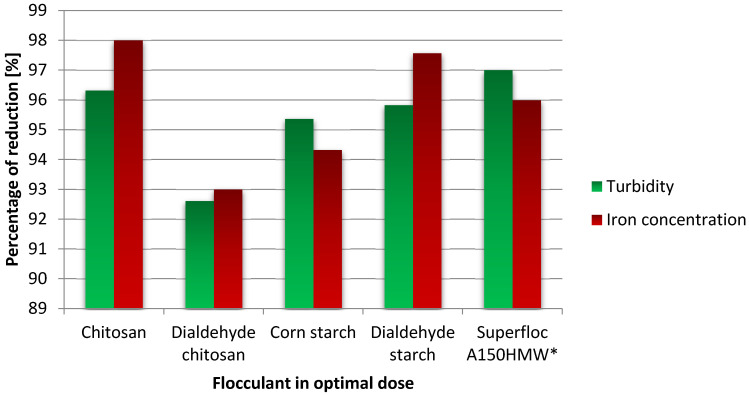
The removal efficiency of turbidity and iron concentration in filter backwash water with flocculants in optimal dose and assistance of 1 mg/L aluminum coagulant (* a reference sample of polyacrylamide flocculant).

Data presented in Figure 12 and Table 5 present show iron and turbidity removal efficiency results according to the optimal dose of bio-based flocculants, comparing the best results for each.

For polysaccharide-based flocculants, the two most likely mechanisms of colloidal particles aggregation have been proposed: charge neutralization and bridging [6,7,50,51]. The first one is associated with the electrostatic attraction of oppositely charged polymers to the surface of the colloidal particles. The bridging is especially effective for long-chain polymers, which can bind multiple particles to form larger aggregates. The Jar Test results indicate that functional groups play a significant role in the flocculation mechanism. The best results were achieved for chitosan, which contains reactive amine groups in macromolecules capable of binding metal ions. The presence of hydroxyls also influences the aggregation process by creating hydrogen bonds. In the case of polysaccharides, specific chemical reactions may also occur between the reactive groups of the polymer and the contaminant particle. One should assume that combining these various interactions occurs simultaneously using polysaccharide flocculants, but the dominant mechanism is bridging.

### 3.4. Biodegradability

An essential feature of flocculants from the point of view of environmental protection is their biodegradability, studied here using respiration tests. The trials examined biochemical oxygen consumption (BOD) of selected polymers and their post-flocculation sludges using the OxiTop measuring device. This method enables observing the biodegradation of organic compounds in different environments over time.

At first, flocculants’ degradation ability was investigated in raw water from groundwater wells located at WTP in Kutno. The results, expressed as oxygen consumption after 7 days and 14 days and biodegradation in %, are presented in Table 6.

The oxygen consumption rate was low in raw water but significant in all tested materials, which means their biodegradability. The BOD in the chitosan and starch-based materials samples was more than 5–15 times higher than the control (endogenous raw water respiration). The analysis shows that the chitosan-based flocculants are more biodegradable than those that are starch-based. After 14 days of degradation, oxygen consumption noted for the oxidized derivatives was almost twice as low as native polysaccharides. The biodegradation (%) recorded in a selected sample of a commercial flocculant (Superfloc A150HMW) after 2 weeks was less than 1%, which indicates its very low susceptibility to the action of microorganisms.

Coagulation and flocculation processes generate large amounts of sludge. Therefore, the next step was to study the decomposition of post-flocculation sludge. Additionally, we checked whether the addition of microorganisms derived from compost and activated sludge from the sewage treatment plant would accelerate the biodegradation rate of post-flocculation sludge.

As the post-flocculation sediments were highly hydrated, their has been evaluated. Furthermore, the sludge enriched with microorganisms from compost and activated sludge was also investigated. Biochemical oxygen demand (BOD) in biodegradation of the post-flocculation sludges (expressed as oxygen consumption per gram dry weight (W/D)) is summarized in Table 7. The BOD values obtained for the sludge from the polysaccharide-based flocculation processes are significantly higher than those obtained for the residual synthetic flocculant (Superfloc A150HMW). The biodegradation process in all samples was intensified considerably by microorganisms from the activated sludge, but only slightly in the presence of microorganisms from the compost. This is due to more bacteria in the activated sludge (30.7 × 10^6^ CFU/mL) than in the compost (2.3 × 10^6^ CFU/g).

**Figure 13:**
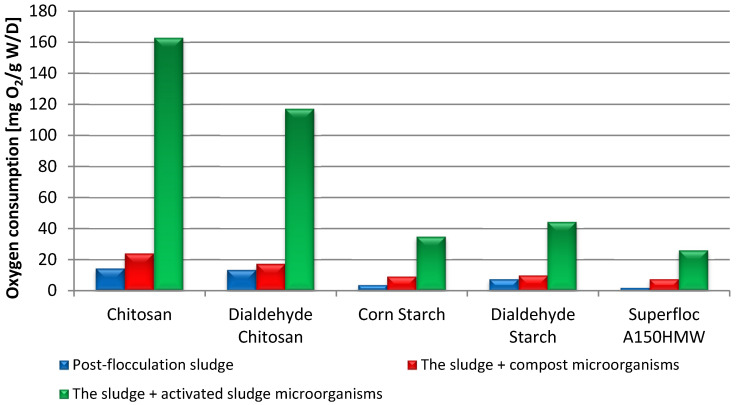
BOD in biodegradation process of post-flocculation sludges after 14 days.

The results of biodegradation for post-flocculation sludge after 14 days are visualized in Figure 13. One can see that the addition of microorganisms from the activated sludge significantly accelerated the decomposition process. It is especially noticeable for chitosan and DCT, for which the BOD increased over 11 and 8 times, respectively. The nitrogen content may explain the greatest biodegradability of chitosan-based flocculants in these samples, which, apart from oxygen, is needed to grow microorganisms. It turned out that DCT, compared to unmodified chitosan, has a specific inhibitory effect on biodegradation, in contrast to DST and starch. Aldehyde groups are toxic to microorganisms due to their reactivity; however, in the case of dialdehyde starch, they are largely involved in the formation of hemiacetals, which explains the differences in the behavior of the polysaccharides tested.

## 4. Discussion

As traces of monomer (acrylamide) exhibiting carcinogenicity and neurotoxicity appear in water treated with polyacrylamide flocculants, the search for alternative non-toxic materials is ongoing. Thus, the international organizations: the Joint Food and Agriculture Organization of the United Nations (FAO)/World Health Organization (WHO) Expert Committee on Food Additives (JECFA) recommend the replacement of harmful for people water treatments aids by safer [13]. These include flocculants based on biopolymers such as chitosan, starch, and their derivatives, e.g., dialdehyde polysaccharides. The materials obtained in this work, the structure and morphology of which were characterized, showed a comparable total iron concentration and turbidity reduction of filter backwash water as commercial polyacrylamide flocculants. The important advantage is efficient biodegradation determined in jar tests.

Oxidation of the studied polysaccharides contributes to morphological changes and modification of their physicochemical properties; in particular, it increases reactivity, improves solubility, reduces the order of macromolecules, and somewhat worsens thermal stability.

According to the literature data [6,7] and the results obtained in this study, the primary mechanism appearing during flocculation with polysaccharide is bridging.

Both polysaccharides and their derivatives have reactive hydroxyl groups that bind impurity molecules. In optimal doses, this effect is the most noticeable. Among the polysaccharide-based flocculants tested, chitosan achieved the best flocculation efficiency. Moreover, the post-flocculation sludge obtained after using this biopolymer was characterized by the highest biodegradation potential.

The best effect of chitosan’s action can be attributed to the presence of amino groups, thanks to which this biopolymer acquires cationic properties favoring electrostatic interactions with the pollutants removed. The remaining acetylamino (in CT) and introduced aldehyde (in DCT and DST) groups can interact with complex iron ions and other positively charged impurities.

To our knowledge, there is little data in the literature on the biodegradation of dialdehyde derivatives of starch and chitosan (although much attention has been paid to the biodegradation of the starting biopolymers [52,53,54,55]).

In the water and other natural environments, microorganisms and their metabolic activity always play a key role in the biodegradation of polymers. To establish the level of metabolic activity of microorganisms during degradation of the flocculants and post-flocculation sludges, the OXI TOP technique was applied. The method is based on measurements of pressure changes resulting from oxygen consumption in a hermetically closed jar containing the sample. Higher BOD in the sample containing the tested material indicates its more intense microbial decomposition, i.e., better biodegradability.

Recently, a paper describing the biodegradability of dialdehyde starch in composites designed for the packaging industry was published [44]. In this work, the inhibiting effect of dialdehyde starch has been found during biodegradation of films modified with food organic compounds, such as caffeine, or ascorbic acid.

Earlier work on thermoplastic dialdehyde starch showed that, due to partial cross-linking of the polysaccharide with aldehyde groups, its biodegradability under controlled composting conditions slows down [56].

Our studies showed a similar effect, i.e., in both DCT and DST, a decrease in the BOD parameter characterizing the biodegradation efficiency is observed compared to unmodified polysaccharides (CT and ST).

There is also a lack of works on their use as flocculants. Only a few articles have been devoted to other modified polysaccharides designed for flocculant production. One of them describes the possibility of using dialdehyde carboxymethyl cellulose grafted on gelatin as a bioabsorbent of organic dyes [57]. In addition, ulvandialdehyde—gelatin hydrogels have been proposed for removing heavy metal ions and dyes from water [58]. Sirvio et al. [59] synthesized cationic cellulose by reaction with Girard’s reagent, after oxidation with periodate. As a result of this modification, a soluble cellulose derivative with a high charge density and an effective flocculating action for calcium carbonate was obtained. Other studies show the possibility of using carboxymethyl cellulose crosslinked by oxidized starch or citric acid as ecological superabsorbents due to the extremely high water absorption [60].

Due to the lack of research on the flocculation properties and biodegradability of dialdehyde starch and chitosan, our work fills this gap. 

## 5. Conclusions

Starch, chitosan and their dialdehyde derivatives obtained by selective cleavage of C2-C3 bonds in glucoside rings constitute an attractive alternative to commercial flocculants based on synthetic polymers. The optimal dose for CT, DCT, ST, and DST was 0.2, 1.0, 1.0, 0.2, respectively. The most effective flocculant turned out to be unmodified chitosan, which was able to remove turbidity and iron ions at the level of commercial polyacrylamide flocculants.

In addition to the expected biodegradability of polysaccharides, the bacterial decomposition of post-flocculation sludge was also found. It is an additional advantage of the proposed solutions for modern, eco-friendly water and wastewater treatment technologies.

Moreover, the materials proposed as flocculants are cheap, and the oxidation method is well known and uncomplicated.

The results indicate considerable potential for applying polysaccharide-based flocculants in the purification process of filter backwash water.

## Figures and Tables

**Table 1:** Commercial polyacrylamide flocculants selected for tests—based on safety data sheets provided by the manufacturer.

Flocculant Name	Ions Fraction Containment	Additives
**Anions polyacrylamide**		
Superfloc A-100PWG	5.4–10.0%	Non
Superfloc A-130 LMW	27.1–36.3%	Non
Superfloc A-150HMW	46.0–59.5%	Non
**Cations polyacrylamide**		
Superfloc 8292	7.3–14.7	Adipic acid 0–5% Citric acid 0–9.9%
Superfloc 8392	No data	Adipic acid 0–5% Citric acid 0–9.9%
Superfloc C492	15.2–25.6%	Adipic acid 0–5% Citric acid 0–9.9%
Superfloc C492HMW	15.2–25.6%	Adipic acid 0–5% Citric acid 0–9.9%
**Nonions polyacrylamide**		
Sedifloc 700AM	-	Non

**Table 2:** Thermal parameters of starch, dialdehyde starch (DST), chitosan, and dialdehyde chitosan (DCT), determined from thermogravimetric curves (Figure 8).

Sample	TGA			DTG		DSC
	∆m_w_^100^ [%]	∆m_t_^600^ [%]	$T_{o}^{TG}$ [°C]	Minor PeaksT_max_ [°C]; R_max_ [%/°C]	Main Degradation Step T_max_ [°C]; R_max_ [%/°C]	Heat [mJ] (Heat Effect); T_max_ [°C]
Starch	9.7	89	296	57; 0.21	316; 2.74	521 (endo); 319
DST	11.9	100	134 199 309	46; 0.33 139; 0.32 251; 0.44	313; 1.94	347 (exo); 314
Chitosan	8.1	63	276	63; 0.17	300; 1.00	626 (exo); 306
DCT	6.5	62	124 313	43; 0.16 128; 0.23	314; 2.70	853 (exo); 314

∆m_w_^100^—loss of water at 100 °C; ∆m_t_^600^—total weight loss in the range of 20–600 °C; $T_{o}^{\mathrm{TG}}$—the onset of degradation steps; T_max_—temperature of peak maximum; R_max_—the rate at the peak maximum.

**Table 3:** The average value of parameters determined for raw filter backwash water during the first and second trials series.

Trials Series	Iron Concentration [mgFe/L]	Turbidity [NTU]	Temperature [°C]	pH
First	25.27	241.29	13.4	7.79
Second	29.67	195.57	13.2	7.70

**Table 4:** Turbidity and total iron concentration in post-flocculated filter backwash water for synthetic flocculant at the optimal dose (removal efficiency ^a,b^).

Flocculant	Optimal Dose [mg/L]	Filter Backwash Water Turbidity [NTU]	Filter Backwash Water Iron Concentration [mg/L]
Superfloc A100PWG	0.5	14.80 (94%)	1.96 (92%)
Superfloc A130	0.2	26.00 (89%)	2.95 (88%)
Superfloc A150HMW	1.0	7.27 (97%)	1.02 (96%)
Superfloc 8292	1.0	11.50 (95%)	1.21 (95%)
Superfloc 8392	1.0	12.40 (95%)	1.31 (95%)
Superfloc C492	1.0	13.80 (94%)	1.95 (92%)
Superfloc C492HMW	1.0	15.40 (94%)	1.90 (92%)
Sedifloc 700AM	0.2	13.90 (94%)	2.10 (92%)

^a^ Turbidity removal efficiency [(T_A_–T_T_)/T_A_] × 100%, where T_A_ and T_R_ are the average turbidity of raw filter backwash water and treated water, respectively. ^b^ Iron removal efficiency [(I_A_ − I_T_)/I_A_] × 100%, where I_A_ and I_T_ is the iron concentration of raw filter backwash water and treated water, respectively.

**Table 5:** Turbidity and iron concentration in FBW after coagulation/flocculation with tested flocculant in optimal dose (removal efficiency).

Flocculant	Optimal Dose [mg/L]	Iron Concentration [mg/L]	Turbidity [NTU]
Chitosan	0.2	0.55 (98%)	6.39 (96%)
DCT	1.0	1.97 (93%)	13.00 (93%)
Corn starch	1.0	1.87 (94%)	9.64 (95%)
DST	0.2	0.81 (98%)	8.04 (96%)

**Table 6:** The results of biodegradation of flocculants in raw water.

Flocculant	Oxygen Consumption [mg O_2_/dm^3^]		Biodegradation % after 14 Days
	7 Days	14 Days	
Chitosan	35.20	43.70	4.05
Dialdehyde chitosan	25.40	28.20	3.59
Corn starch	18.30	26.80	2.62
Dialdehyde starch	5.60	14.10	2.55
Superfloc A150HMW	5.60	8.50	0.88
Control sample ^1^	2.40	2.80	-

^1^ Raw groundwater collected at the WTP in Kutno.

**Table 7:** BOD in biodegradation of the post-flocculation sludges.

Flocculant	Oxygen Consumption [mg O_2_/g W/D]					
	Post-Flocculation Sludge		The Sludge + Compost Microorganisms		The Sludge + Activated Sludge Microorganisms	
	7 Days	14 Days	7 Days	14 Days	7 Days	14 Days
Chitosan	11.2	14.4	13.9	24.0	103.7	162.9
DCT	9.6	13.4	10.3	17.3	49.6	117.2
Corn Starch	1.8	3.7	3.7	9.2	19.3	34.8
DST	4.9	7.4	6.2	9.9	31.1	44.3
Superfloc A150HMW	0	1.8	5.9	7.4	19.8	26.0

## Data Availability

Not applicable.
